# Retrospective Study of Morton’s Neuroma: Clinical, Paraclinical, and Therapeutic Assessment of 10 Cases

**DOI:** 10.7759/cureus.75968

**Published:** 2024-12-18

**Authors:** Manare Jaai, Haytam Mellouki, Ahmed Amine El Oumri

**Affiliations:** 1 Physical Medicine and Rehabilitation, University Hospital Center Mohammed VI Oujda Morocco, Oujda, MAR; 2 Physical Medicine and Rehabilitation, Faculty of Medicine of Oujda, Mohammed VI University Hospital of Oujda, Mohammed First University of Oujda, Oujda, MAR

**Keywords:** corticosteroid injection, extracorporeal shock wave, forefoot pain, metatarsalgia, mulder's sign, radio frequency, surgical nerve decompression, tingling or numbness, ultrasound imaging

## Abstract

Background

Morton’s neuroma is a common cause of forefoot pain, typically occurring in the third metatarsal space and characterized by symptomatic nerve compression. This condition often leads to significant functional impairment, affecting weight-bearing activities and limiting appropriate footwear due to pain and discomfort.

Objective

This study aims to evaluate the outcomes of conservative interventional treatment for Morton’s neuroma, specifically focusing on corticosteroid injections.

Methodology

We reviewed 10 cases of Morton’s neuroma in the field of physical medicine and rehabilitation. The average age of the patients was 45 years, with a higher prevalence among females. Diagnoses were confirmed through clinical assessment and ultrasound imaging.

Results

All 10 patients (100%) received conservative interventional treatment with corticosteroid injections. This approach effectively alleviated symptoms in 9 out of 10 patients (90%). Surgical options were considered only if symptoms did not improve with this treatment.

Conclusions

All 10 patients (100%) were treated with corticosteroid injections, which alleviated symptoms in 9 out of 10 patients (90%). Surgical options were considered only if this treatment was insufficient. This analysis highlights the effectiveness of this approach as a first-line management strategy for Morton’s neuroma.

## Introduction

Morton’s neuroma is a benign disorder that occurs in the third intermetatarsal space of the foot and is characterized by degeneration and fibrosis of the plantar nerve [1]. First described by physician Thomas G. Morton in 1876 [2], this condition results from the compression of the common and proper digital nerves, leading to symptoms such as burning, tingling, or numbness.

Epidemiological studies suggest that Morton’s neuroma affects approximately 30% to 33% of individuals with foot pain and is more prevalent in women aged 25 to 55 years [3]. Diagnosis is typically based on a comprehensive clinical evaluation and detailed patient history [4], with confirmation often supported by imaging modalities, including radiography to exclude other pathologies and ultrasound and magnetic resonance imaging [5].

Treatment options include conservative measures such as nonsteroidal anti-inflammatory drugs (NSAIDs), physical therapy, orthotic devices, corticosteroid injections, sclerosing agents, shock wave therapy, and radiofrequency ablation (RFA), which are effective in approximately 85% of cases [6]. Surgical intervention is considered when conservative treatments fail to provide adequate relief [7].

The aim of this study is to review the clinical presentations, diagnostic methods, and treatment options for Morton’s neuroma, drawing from both our clinical experience and a comprehensive review of the current medical literature.

## Materials and methods

Study design and setting

This retrospective study was conducted at CHU Mohammed VI, Oujda, in the Department of Physical Medicine and Rehabilitation, from May 13, 2023, to October 16, 2023. It included 10 patients diagnosed with Morton’s neuroma, each evaluated and managed according to the department’s clinical protocols.

Inclusion criteria

Patients were eligible for inclusion based on their management within the Department of Physical Medicine and Rehabilitation and fulfillment of the diagnostic criteria for Morton’s neuroma. The diagnosis was confirmed through a clinical evaluation, including the Mulder test, and corroborated by ultrasound imaging. Furthermore, all patients must have undergone corticosteroid injections administered according to the facility’s established treatment protocol.

Exclusion criteria

Patients were excluded if their medical records were incomplete, specifically lacking essential information such as detailed diagnostic results, treatment outcomes, or follow-up data necessary for a thorough analysis.

Data collection and analysis

Medical records were reviewed using a standardized data collection form to ensure systematic gathering of patient demographics, clinical details, diagnostic results, and treatment information. Data extraction was performed by the study author to guarantee accuracy. Descriptive and statistical analyses were subsequently conducted to evaluate treatment effectiveness and patient outcomes, aligning with the research objectives.

## Results

**Figure 1:**
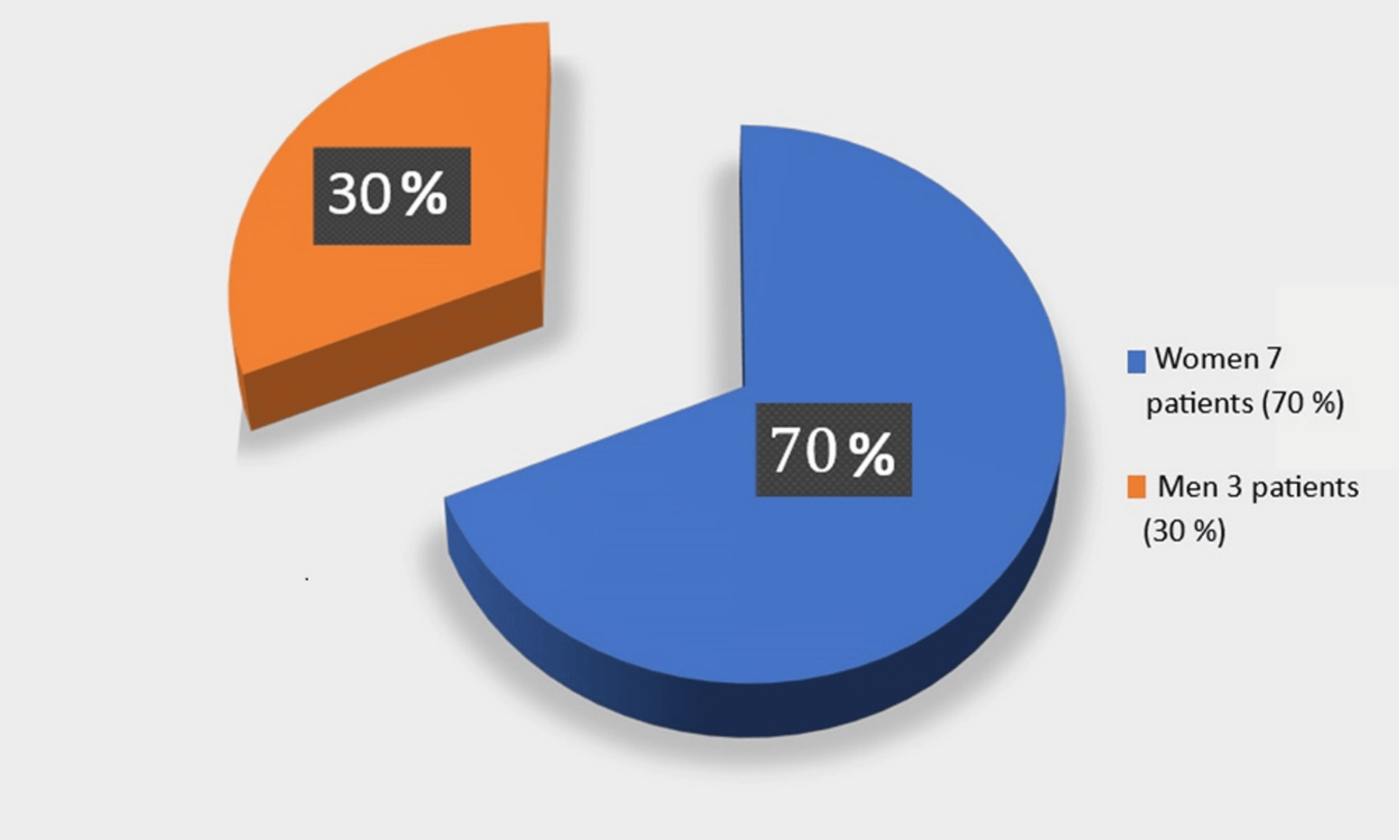
Distribution of patients by gender. This figure illustrates the gender distribution of patients in our study, highlighting the predominance of women (7, 70%) among the cohort.

In our study, the average age of the patients was 45 years, with a range from 26 to 65 years. The gender distribution included seven women (70%) and three men (30%), resulting in a male-to-female ratio of 0.43. As depicted in Figure 1, the majority of our patients were women, suggesting a higher susceptibility to Morton’s neuroma in females, as evidenced by the significant representation of women in our cohort.

**Figure 2:**
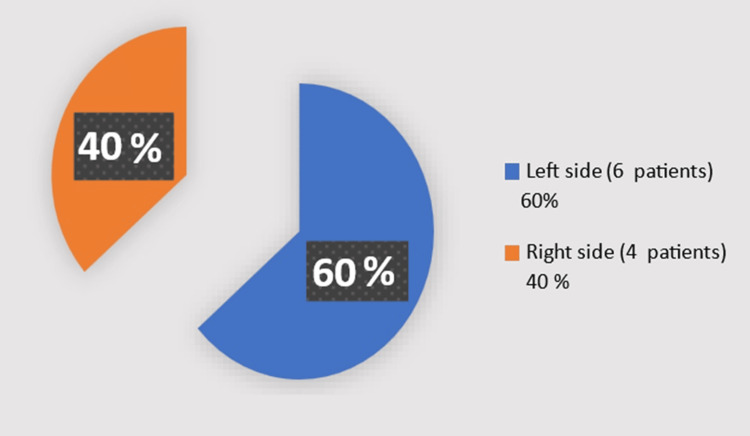
Distribution of patients according to the affected side. The illustration highlights the laterality of Morton’s neuroma in our study, revealing that the left side was more frequently affected (6, 60%) than the right (4, 40%).

In this cohort, the majority of cases were on the left side, constituting 60%, while the right side accounted for 40%. As indicated in Figure 2, this distribution highlights the tendency for Morton’s neuroma to more frequently affect the left side.

**Figure 3:**
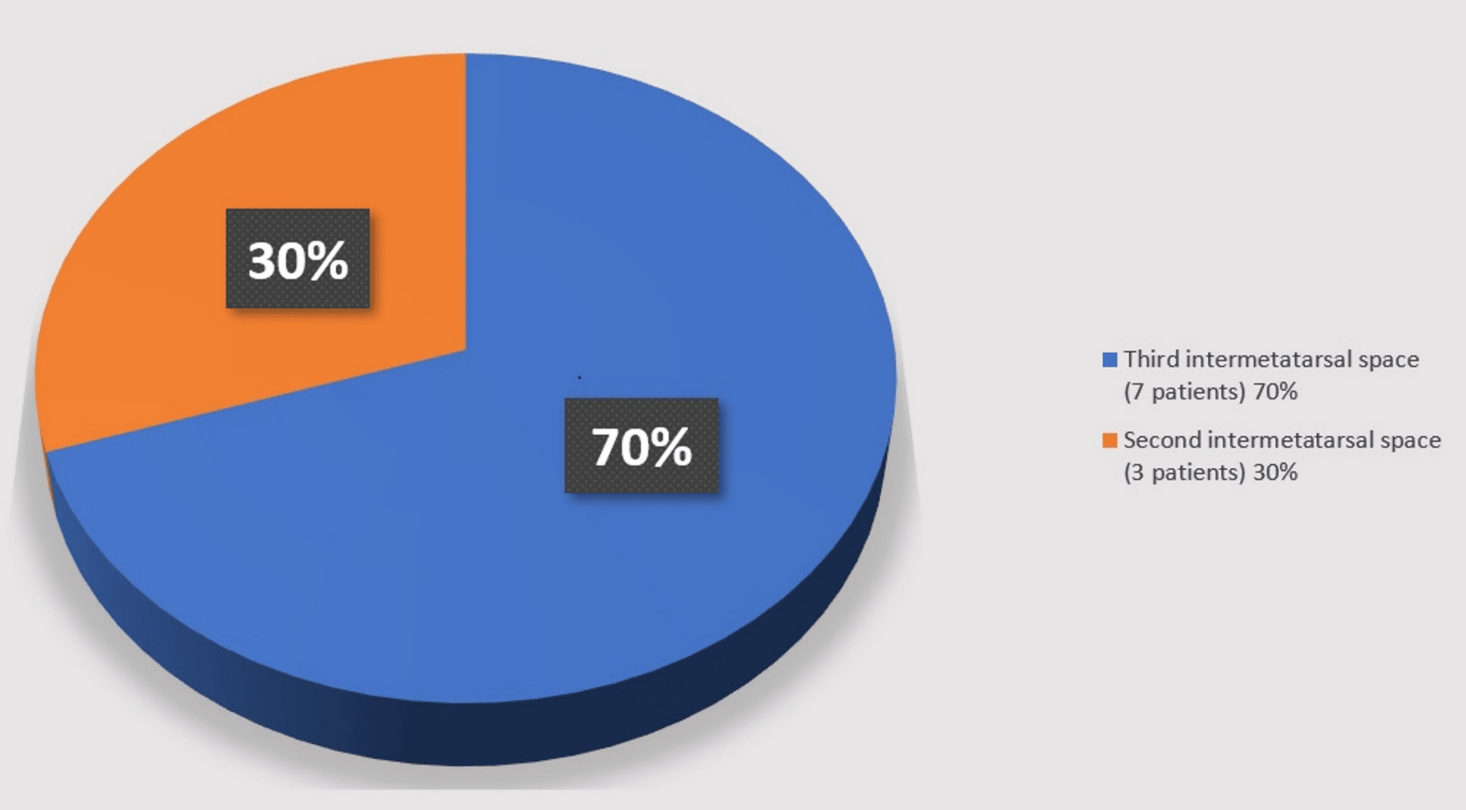
Distribution of patients by intermetatarsal space. The diagram illustrates the distribution of Morton’s neuroma cases by intermetatarsal space, showing that 70% of cases occur in the third intermetatarsal space, while 30% are found in the second.

In our analysis, the third intermetatarsal space was affected in seven cases, representing 70%, while the second intermetatarsal space was involved in three cases (30%). As shown in Figure 3, this distribution highlights the predominance of involvement in the third intermetatarsal space.

**Figure 4:**
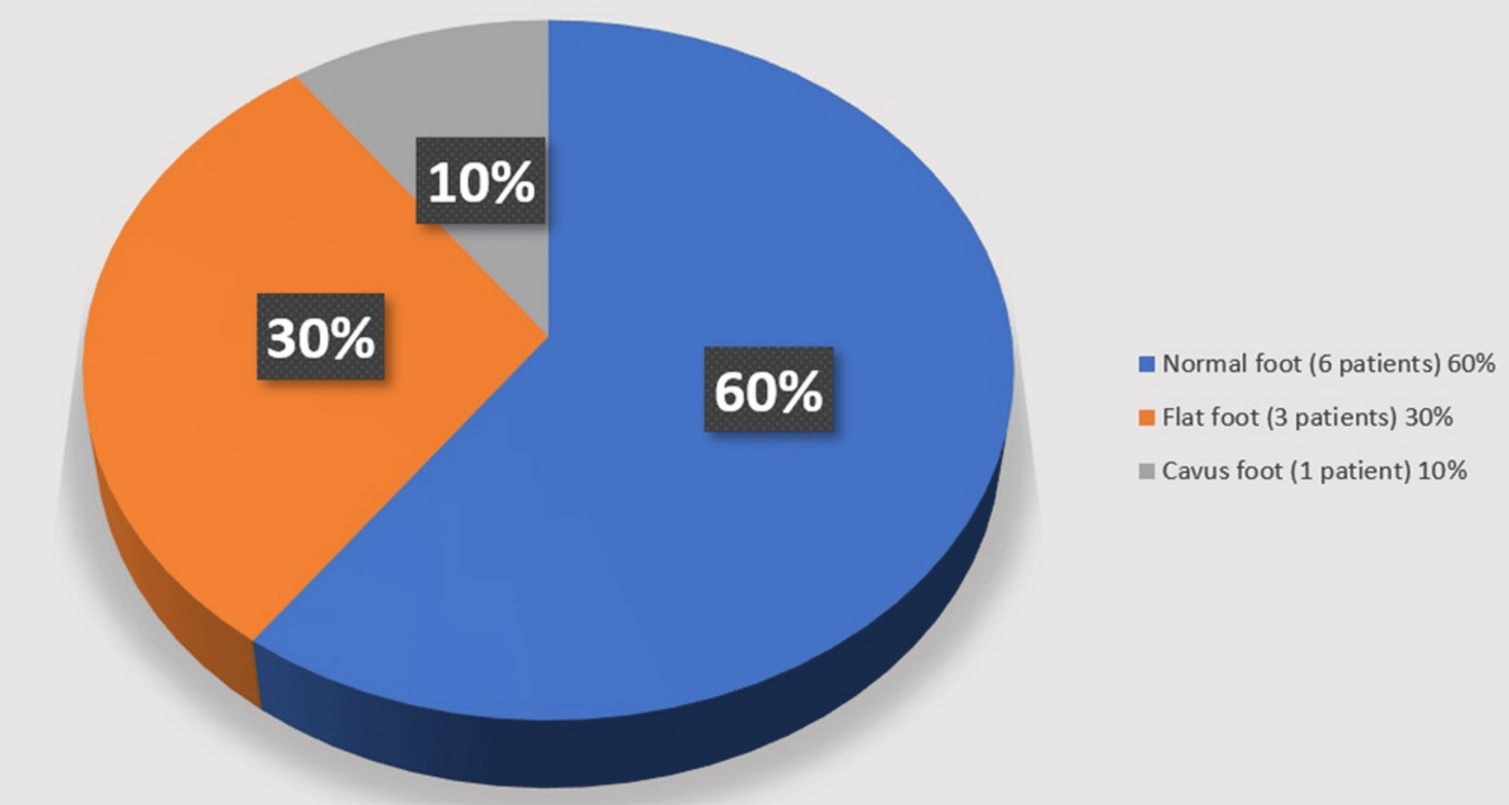
Classification of patients by foot type. The chart outlines the distribution of foot types among the affected feet of the patients, with six (60%) presenting a normal foot, three (30%) having a flat foot, and one (10%) showing a cavus foot, emphasizing the predominance of a normal foot structure.

In this assessment, the prevalence of foot types revealed that six patients had a normal foot (60%), three patients were identified with a flat foot (30%), and one patient exhibited a cavus foot (10%), as presented in Figure 4. This highlights the predominance of normal foot morphology within the evaluated population.

Concerning patient histories related to factors contributing to foot conditions. Six patients (60%) reported a history of wearing tight shoes, while four patients (40%) had experienced foot trauma. Among those with preexisting conditions, three patients (30%) had additional medical issues such as diabetes and high blood pressure. Additionally, two patients (20%) were classified as obese, and one patient (10%) had a history of rheumatic disease, as presented in Table 1. This highlights the diverse factors that may contribute to the development of Morton’s neuroma.

**Table 1:** Medical backgrounds of patients. This table summarizes factors contributing to foot conditions. Sixty percent of patients reported wearing tight shoes, and 40% had foot trauma. Among those with preexisting conditions, 30% had issues like diabetes, 20% were obese, and 10% had rheumatic disease.

Medical background	Number of cases	Percentage
Tight shoes	6	60%
Foot trauma	4	40%
Diabetes	3	30%
Obesity	2	20%
High blood pressure	3	30%
Tobacco	3	30%
Rheumatic disease	1	10%

In this clinical study, pain was the primary reason for consultation among all patients, leading to significant activity limitations, including reduced walking distances and changes in footwear. Clinical examination revealed that all patients experienced discomfort upon palpation of the metatarsophalangeal (MPT) joint. Additionally, swelling was noted in six cases (60%), decreased sensitivity was observed in five cases (50%), and tenderness was documented in one case (10%). As detailed in Table 2, these findings demonstrate the impact of the condition on patients' daily activities and overall quality of life.

**Table 2:** Clinical examination results in our series. The table outlines that every patient reported pain and restricted activities. It further indicates that six (60%) experienced swelling, five (50%) suffered from decreased sensitivity, and one (10%) had tenderness, highlighting the overall impact of these symptoms on daily living. MPT, metatarsophalangeal

Symptomatic and clinical examination findings	Number of cases	Percentage
Pain	10	100%
Limitation of activities	10	100%
Adjacent joint swelling	6	60%
Decreased sensitivity in the MPT joint	5	50%
Metatarsophalangeal joint tenderness	1	10%

**Figure 5:**
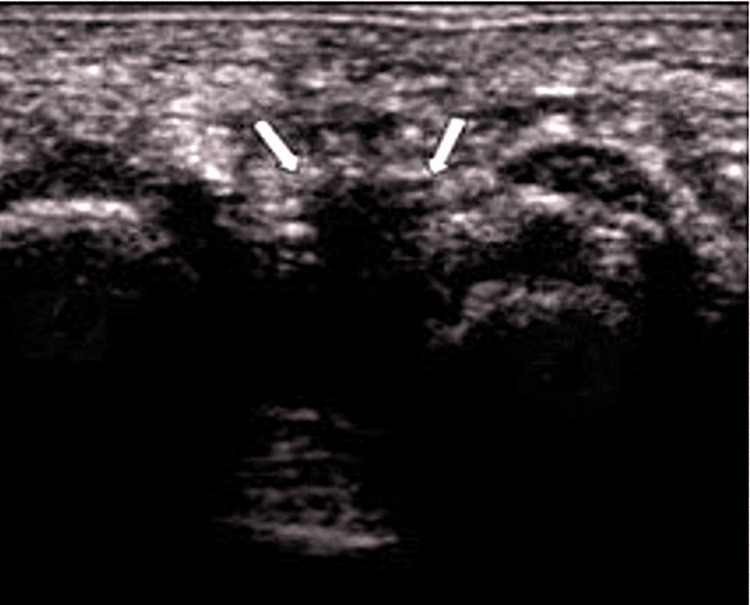
Ultrasound image showing Morton's neuroma in a patient. Image Credit: Manare Jaai The image presents a 7 mm hypoechoic ovoid lesion along the longitudinal axis between the third and fourth metatarsals. These findings are crucial for diagnosing the underlying conditions affecting the patient.

In the paraclinical assessment, each patient underwent a foot ultrasound, which revealed an abnormality between the third and fourth metatarsals. The characteristics of this finding, including size and echogenicity, are significant for diagnosing underlying conditions. Overall, these ultrasound findings are crucial for guiding further clinical evaluation and management, ensuring timely diagnosis and appropriate treatment, as described in Figure 5.

All patients received medical management, including analgesics, anti-inflammatories, and plantar orthoses with retro-capital support. Rehabilitation was also part of the treatment plan. However, due to limited improvement with these measures, local corticosteroid injections under ultrasound guidance were recommended for all patients, demonstrating significant efficacy in treating Morton's neuroma, as illustrated in Table 3.

**Table 3:** Treatment approach for patients with Morton's neuroma in our series. This table outlines the treatment protocol for Morton's neuroma, starting with analgesics, anti-inflammatories, and orthoses. Though initially ineffective, it highlights the key role of ultrasound-guided corticosteroid injections in significantly improving symptoms.

Type of treatment	Number of cases	Percentage
Analgesic and anti-inflammatory	10	100%
Orthosis with retro-capital support	10	100%
Physical therapy	10	100%
Ultrasound-guided local corticosteroid infiltration	10	100%

In assessing the effectiveness of the treatment protocol, a comprehensive evaluation of patient satisfaction was conducted using a scoring system designed to gauge various aspects of the treatment experience. As indicated in Table 4, the results show that five patients (50%) were very satisfied, three patients (30%) were satisfied, and two patients (20%) were somewhat satisfied. Notably, no patients reported being dissatisfied with the treatment. Additionally, no complications, such as infection, hematoma, or delayed healing, were detected. The criteria assessed included pain intensity, footwear modification, and activity limitation, providing a thorough evaluation of the treatment's impact.

**Table 4:** Treatment outcomes for patients with Morton’s neuroma. This table displays treatment satisfaction, with five (50%) patients reporting very satisfied, three (30%) satisfied, two (20%) somewhat satisfied, and none dissatisfied, while assessing pain intensity, footwear modification, and activity limitation.

Satisfaction index	Number of cases	Percentage
Very satisfied	5	50%
Satisfied	3	30%
Somewhat satisfied	2	20%
Dissatisfied	0	0%

In this study, we assessed the demographic data of all patients diagnosed with Morton’s neuroma. Table 5 summarizes key information, including age, gender, affected side, foot type, and relevant medical history.

**Table 5:** Demographic data of patients with Morton’s neuroma. The table presents the demographic characteristics of the 10 study patients, highlighting their age, gender, affected side, and relevant medical histories, for a comprehensive cohort overview.

Patient ID	Age (years)	Gender	Affected side	Affected intermetatarsal space	Foot type	Medical history
1	26	Female	Right	3rd	Normal	Foot trauma, tight shoes
2	34	Female	Left	3rd	Flat	Foot trauma
3	38	Female	Left	3rd	Normal	Tight shoes
4	40	Female	Left	2nd	Flat	Tight shoes, diabetes
5	45	Female	Left	3rd	Normal	Foot trauma, tight shoes, obesity, high blood pressure
6	45	Female	Right	2nd	Normal	Rheumatic disease, tight shoes, diabetes
7	50	Male	Right	3rd	Cavus	Diabetes, high blood pressure, tobacco
8	52	Female	Right	3rd	Normal	Obesity, diabetes, tight shoes
9	58	Male	Left	3rd	Normal	Tobacco, high blood pressure
10	65	Male	Left	2nd	Flat	Foot trauma, tobacco

## Discussion

Morton’s neuroma is a neuropathy that most commonly arises in the third intermetatarsal space of the forefoot, primarily caused by pressure and irritation at the plantar aspect of the transverse intermetatarsal ligament [8]. First described by Thomas G. Morton in 1876, this condition presents as a distinct and painful pathology affecting the metatarsal region [9].

The pathophysiology of Morton’s neuroma is commonly attributed to chronic mechanical trauma, leading to perineural fibrosis and subsequent fibrous enlargement of the affected nerve. This process involves vascular changes such as arterial degeneration, along with endoneurial edema and axonal degeneration [10].

Morton’s neuroma is notably more prevalent among middle-aged women, with an incidence at least five times higher in females compared to males [11]. Although the exact prevalence remains poorly defined, studies suggest a higher predisposition in women. While bilateral involvement is uncommon, the occurrence of multiple neuromas in different intermetatarsal spaces on the same foot is relatively frequent [12].

Diagnosing Morton’s neuroma primarily relies on clinical evaluation while excluding other pathologies. The condition is characterized by localized forefoot pain, along with burning, numbness, and tingling in the third and fourth toes. Symptoms are often exacerbated by weight-bearing activities and direct pressure on the interdigital nerve. Relief is typically achieved through rest and avoidance of aggravating footwear. During physical examination, a palpable mass may be detected in approximately one-third of patients, often accompanied by Mulder’s sign [13-14].

Although Morton’s neuroma is primarily diagnosed through clinical evaluation, imaging techniques such as radiography, ultrasonography, and magnetic resonance imaging (MRI) can aid in the diagnostic process.

Radiographic imaging is primarily used to exclude differential diagnoses such as avascular necrosis, osteoarthritis, Freiberg’s disease, and stress fractures. Additionally, this assessment may reveal splaying of the metatarsals or a small soft-tissue shadow related to the lesion, although these findings are not definitive for diagnosis [15]. In the studies by Esling et al., all patients underwent weight-bearing X-rays of the foot, including both anterior-posterior and lateral views, to rule out these conditions [4].

Ultrasonography has become a valuable diagnostic modality due to its cost-effectiveness compared to MRI and its applicability in various clinical settings. Morton’s neuroma typically appears as a hypoechoic, ovoid mass oriented parallel to the long axis of the metatarsals and is best visualized in the coronal plane [16-17]. Kankanala and Jain found that ultrasonography has a 91.67% probability of accurately diagnosing plantar intermetatarsal neuroma. Their study reported a sensitivity of 91.48%, perfect specificity, and a positive predictive value of 100%. However, the negative predictive value was 20% [18]. In our series, all 10 patients underwent ultrasonography to confirm the diagnosis.

MRI is useful for excluding other masses or pathologies in the area but is generally not required for diagnosing Morton’s neuroma. This condition typically appears as a mass with low signal intensity on both T1- and T2-weighted sequences, located between or just distal to the MPT joints [14]. The fibrous content of the neuroma contributes to its distinct appearance, aiding in the differentiation from conditions such as schwannomas or intermetatarsal bursae, which are typically hyperintense on T2-weighted images [19]. 

Initial treatment of Morton’s neuroma typically emphasizes conservative strategies. These involve reducing weight-bearing activities, engaging in physical therapy to strengthen the foot and improve gait mechanics, and modifying footwear, such as low-heeled shoes and metatarsal padding to relieve forefoot pressure. Orthotic insoles, whether custom-made or over-the-counter, help provide arch support and evenly distribute pressure across the foot. Additionally, pharmacologic management, including the use of oral NSAIDs, is employed to reduce inflammation and manage pain [20].

Infiltrative options also play a significant role in treating Morton’s neuroma. These include corticosteroids, alcohol, phenol [21], botulinum toxin [22], and capsaicin [23]. Among these treatments, corticosteroid injections are frequently employed due to their effectiveness in providing symptomatic relief, with improvements in outcome measures typically observed after 12 months [23-24]. A study by Markovic et al. [25] reported total satisfaction in 38% of patients at nine months following a single injection. In our series, all 10 patients (100%) received corticosteroid treatment and experienced substantial symptom improvement.

Furthermore, extracorporeal shock wave therapy has also emerged as a valuable treatment option. Administered on an outpatient basis, it has demonstrated significant improvement in pain scores for up to 12 weeks, as reported in a randomized, placebo-controlled, double-blind trial involving 25 feet [26].

Another promising treatment modality is RFA, first introduced by Finney et al. in 1989 [27]. A study by Moore et al. [28] found that 83% of patients achieved complete symptom relief within one month of treatment, with only one out of 29 patients requiring subsequent surgical excision. This highlights the effectiveness of RFA as a minimally invasive option for managing Morton’s neuroma.

When conservative management fails to provide adequate symptom relief, surgical approaches for treating Morton’s neuroma are indicated. These encompass nerve decompression techniques, including neurolysis or translocation of the affected interdigital nerve, as well as neurectomy, which involves the complete resection of the affected nerve segment. Common surgical techniques consist of dorsal and plantar longitudinal incisions, along with approaches like transverse plantar, web-splitting, and Y-incision. The plantar approach provides direct access to the lesion but may lead to painful scar hypertrophy due to its location in the weight-bearing forefoot area. In contrast, the dorsal approach involves sectioning the intermetatarsal ligament, which can avoid a painful weight-bearing scar but may risk missing nerve branches oriented toward the plantar side, potentially increasing the risk of recurrence [29].

Finally, Barrett and Pignetti described endoscopic decompression of the intermetatarsal nerve in a cadaveric study, suggesting potential benefits such as smaller incisions, faster recovery, reduced postoperative pain and swelling, and a lower incidence of hematoma and infection [30]. This technique exemplifies the ongoing evolution of treatment options for Morton’s neuroma, aiming to improve patient outcomes while minimizing complications.

This study has several limitations. The retrospective design may introduce selection bias and limit the generalizability of the findings. The small sample size of 10 patients, while informative, restricts the statistical power of the results. Variations in treatment protocols and follow-up durations can also impact outcomes and should be considered when interpreting the findings. Additionally, the reliance on self-reported satisfaction and symptom relief may introduce subjective bias. Future studies with larger sample sizes and more robust prospective designs are warranted to validate these results and enhance our understanding of Morton’s neuroma management.

## Conclusions

This study identifies Morton’s neuroma as a prevalent neuropathic condition, particularly affecting the third intermetatarsal space in middle-aged women. Diagnosis relies primarily on clinical evaluation, with ultrasound serving as a valuable confirmatory tool. The pathogenesis is likely linked to chronic mechanical stress or repetitive trauma.

Our findings indicate that corticosteroid injections are effective as a first-line intervention, providing significant symptomatic relief. Alternative treatments, such as sclerosing agents, shock wave therapy, and radiofrequency ablation, are available for patients unresponsive to conservative measures, emphasizing the need for further research in larger cohorts. Surgical options are considered when conservative management fails, typically involving a dorsal approach for nerve excision or decompression.
